# A New Automatic Foot Arch Index Measurement Method Based on a Flexible Membrane Pressure Sensor

**DOI:** 10.3390/s20102892

**Published:** 2020-05-20

**Authors:** Tao Zheng, Zhiyong Yu, Jin Wang, Guodong Lu

**Affiliations:** State Key Laboratory of Fluid Power and Mechatronic Systems, Zhejiang University, Hangzhou 310027, China; zhengtao2955@zju.edu.cn (T.Z.); k1062@zju.edu.cn (Z.Y.); lugd@zju.edu.cn (G.L.)

**Keywords:** foot arch index, flexible membrane pressure sensor, plantar pressure, image processing, 8-neighborhood correlation pixel, row element association, high arch foot, flat foot

## Abstract

The foot arch index is an important index to evaluate the health of human feet and the biomechanics line, aiming at addressing the shortcomings of the low efficiency and slow speed of manual foot arch index measurement; in this work, an automatic foot arch index measurement method based on a flexible membrane pressure sensor was proposed. The distribution of plantar pressure data was obtained from the flexible membrane pressure sensor and converted into a digital image. The 8-neighborhood correlation pixel method was proposed to remove the interference of isolated noise points. In order to remove the toes’ data without affecting the foot sole data, the row element association algorithm was proposed. The front and back endpoints of the foot were automatically located to obtain the foot length, and the foot arch index was also automatically obtained based on the foot arch pressure area. Whether it was a high arch foot, flat foot or normal foot, the method proposed in this paper could accurately and quickly distinguish them. The prototype was developed, and its feasibility and validity were verified by a series of tests.

## 1. Introduction

The human foot has a complex multi-link structure, which plays an important supporting role in daily activities. The foot plays a role of cushioning and shock absorption during exercise, and the normal arch can keep the balance of the body. Some studies have shown that foot problems can lead to a variety of brain, spine, knee, internal organ and other diseases [1,2,3,4], Furthermore, understanding the shape of an individual’s foot or foot-arch is necessary to provide direct and useful information, not only for clinical and rehabilitative purposes, but also for designing personalized and comfortable footwear [5,6,7]. Thus, research on the foot arch index has a positive and important guiding role for our health.

A normal foot arch has a positive buffering effect on the brain, spine, knee and other visceral organs. The direct factor that determines the cushioning effect is the shape of the foot arch. Generally, the arch mainly includes three types: high arch feet, flat feet and normal feet [8,9,10]. A high arch foot is usually caused by an increase in the longitudinal arch of the front foot. When walking, the force point of the foot is usually only the toe and heel. People with high arch feet usually have better jumping ability, but because the effective force area of the foot is smaller, the pressure of the foot is larger, so it is not suitable for walking for a long time or a long distance. The intuitive manifestation of the flat foot shape is that the arch of the foot collapses or disappears when standing. The main reason is the tendons and muscles cannot provide sufficient support and it is mainly due to acquired factors. Flat feet are usually stressed on the whole soles of the feet; the buffering effect is seriously weakened when walking. They will cause the biomechanics line to skew and the knees’ abrasion, which is also not suitable for long time or long distance walking. The normal arch foot has a positive buffering and protective effect on the whole body and protects the entire body from a variety of diseases. However, the number of people with arch defects is increasing nowadays; therefore, a more accurate, faster, more convenient and more intelligent measurement method for the arch index and an effective treatment method for arch defects should have a positive impact on the improvement of people’s quality of life, in which a fast and accurate measurement of foot arch shape defects is an important prerequisite for correct treatment [11,12].

The existing arch index measurement methods mainly include two types of contact measurement and non-contact measurement [13,14,15,16,17]. Contact measurement is the manual measurement of foot parameters using some common measuring instruments, for example, caliper measurement. Non-contact measurement is mainly based on optical imaging or a plantar pressure sensor. The main idea of this approach is that a camera, scanner or sensor captures the foot surface data and measurement is done by analyzing the captured visual or sensor data using hardware and software technologies [17,18,19].

The manual measurement method is less efficient and time consuming; the accuracy of the measurement is dependent on the measurement skill of the experimenter, and the reliability and repeatability are, thus, usually poor. The optical image scanning measurement approach, with the progress of science and society’s development, now can be used in a variety of applications and scenarios: the measurement of human body parts, hand posture recognition, face recognition, and 3D human body reconstruction [20,21,22,23], etc. However, it usually needs to carry out three-dimensional modeling, the computational-cost is high, and the cost of the system is also very high. The method based on plantar pressure measurement is not only low in price but also low in computational cost. However, most of the plantar pressure distribution-based measurement methods use several single point force sensors to estimate the pressure distribution; they are low in accuracy and still need manual intervention to complete the measurement [24,25], which is still inefficient. In recent years, flexible membrane pressure sensors have been widely used, but there are few studies on automatic arch index measurement based on flexible membrane pressure sensors; meanwhile, the market demand for an efficient, fast and low cost automatic arch index measurement is urgent. Therefore, it is of great clinical value to design a flexible membrane pressure sensor-based automatic measurement method for the arch index.

In this work, an intelligent automatic foot arch index measurement method based on a flexible membrane pressure sensor was proposed. It can automatically realize noise reduction and measurement with high efficiency and accuracy. The foot arch index calculation method mainly includes three types: measurement based on the height of the foot arch, measurement based on the foot arch pressure area and measurement based on the arch width. The arch index based on the arch height is obtained by the ratio of the arch height to the foot length. The arch index based on the pressure area is obtained from the ratio of the arch area to the whole foot area (excluding the toes), and the arch index based on arch width is obtained by the ratio of the arch width to the foot length [8,11,26,27,28]. The calculation method used in this work is the ratio of foot arch pressure area to the whole foot area (excluding the toes).

In the following part of this paper, we describe the algorithm principle and framework of the proposed method. A test prototype was designed, and a series of comparative experiments were carried out to verify the validity of the method.

## 2. Algorithm Principle

**Figure 1:**
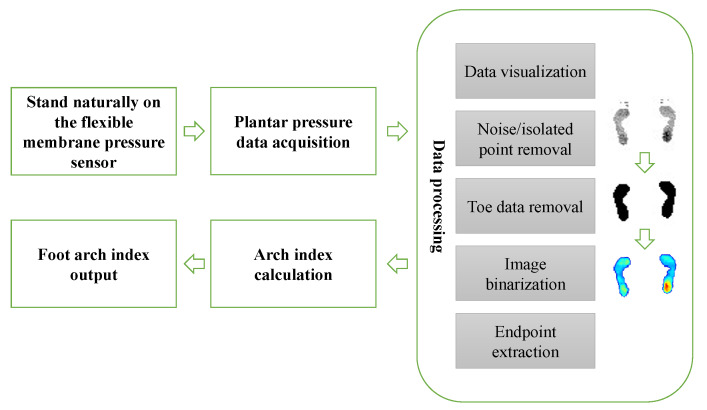
The framework of the proposed algorithm.

This part mainly introduces the principle and detailed framework of the proposed automatic arch index measurement method. The detailed flow of the algorithm is shown in Figure 1, which mainly includes plantar pressure data acquisition, data visualization, isolated noise point removal, toe data removal, image binarization, key point extraction and arch index calculation.

### 2.1. Calculation Method for Foot Arch Index

As mentioned above, the arch index calculation method mainly includes three types. Among them, the representative method is the arch index obtained from the ratio of the arch pressure area to the whole foot pressure area. This method is adopted in this work. Equation (1) shows the calculation method, where β is the arch index,

(1) $$\beta =s_{2}/(s_{1}+s_{2}+s_{3}).$$

**Figure 2:**
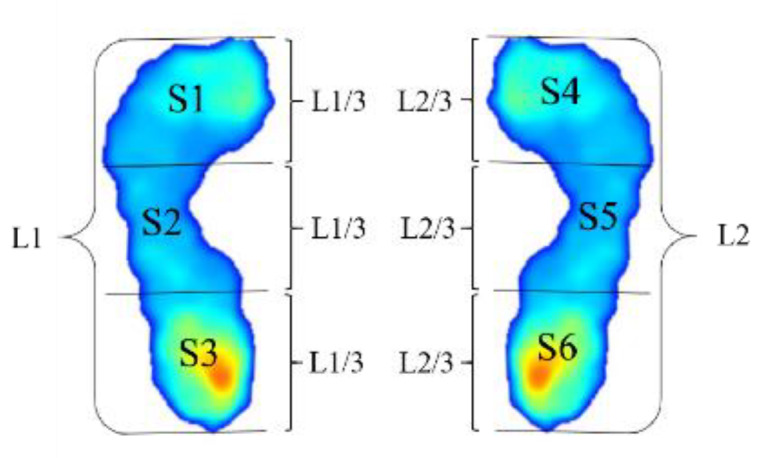
The corresponding area definition of the arch index measurement.

As shown in Figure 2, L1 and L2 are the lengths of the left and right feet, respectively. $s_{1}$ is the effective pressure area of the first one third of the foot length, $s_{2}$ represents the effective arch pressure area of the middle one third of the foot, and $s_{3}$ is the effective pressure area of the last one third of the length.

### 2.2. Parameters of Used Flexible Membrane Pressure Sensor

**Figure 3:**
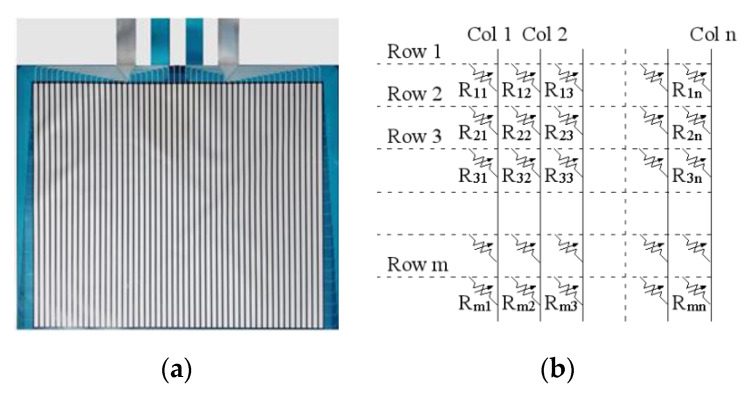
Flexible membrane pressure sensor. (**a**) Flexible membrane pressure sensor. (**b**) Equivalent circuit of sensing points.

In this work, a flexible membrane pressure sensor is used to collect the plantar pressure data; the effective size of the flexible membrane pressure sensor is 36.5 cm in length and 30.5 cm in width. The total sensing points number is 2288, with 44 rows and 52 columns. The output voltage range is 0 V–3.2 V. Shown in Figure 3a is the flexible membrane pressure sensor, and Figure 3b is the equivalent circuit of sensing points. Each sensing point is a force sensing resistor made of piezoresistive materials. It exhibits a decrease in resistance with an increase in the force applied to the surface of the sensor. The flexible membrane resistance sensor is fabricated with the double-sided polyester substrate respectively by the layers including the printed silver electrode one, the stress sensitive one, the insulation one (option), and the glue one. Then, on the layer of the substrate package, there were two terminal electrodes punched with the data acquisition circuits. It allows the forces of the static force and dynamic forces between two surfaces. When applying force on the effective area of the sensor, a decrease in the resistance between two leads of the sensor will be induced, and the larger the applied force, the smaller the resistance, so that the measured voltage is different. The obtained voltage is stored in a 1 × 2288 array and sent to the computer via a USB cable. Special attention should be paid to the fact that before testing, calibration has to be done; if the sensitivity threshold voltage value is too low, too much noise will be considered as valid data, and if the threshold is set too high, a large number of effective data will also be filtered out. 

### 2.3. Sensor Data Acquisition and Processing

The array with the stored pressure data acquired from flexible membrane pressure sensor is transformed into a 44 × 52 matrix, which is easily visualized and convenient for subsequent processing. The range of the obtained 2288 pressure data is x∈[0,3.2]; by using Equation (2), the value of each point will be mapped to a range of 0 to 255 to form a grayscale image.

(2) gray=x/3.2×255.

**Figure 4:**
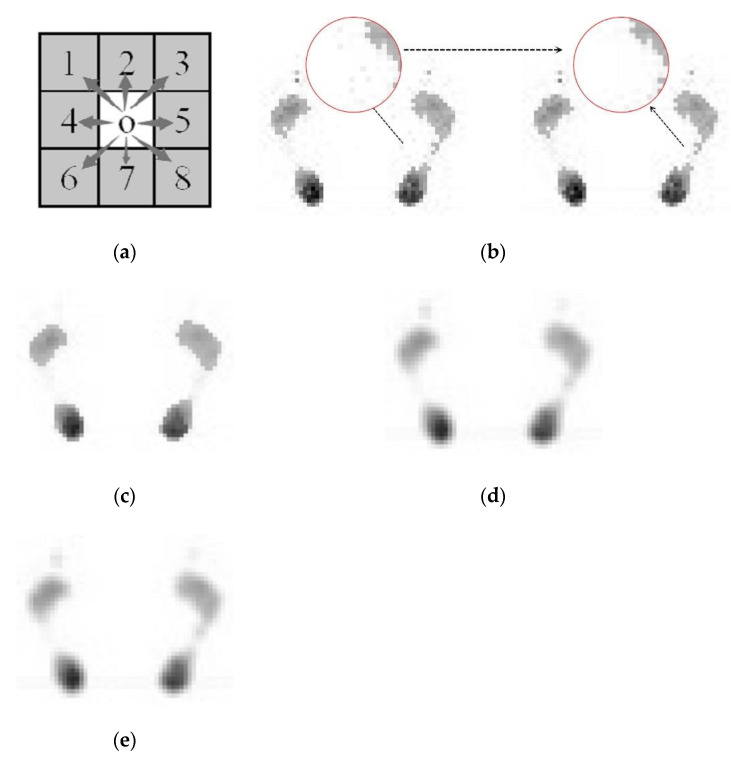
(**a**) shows the definition of the 8-neighborhood correlation pixel and (**b**) is the original plantar pressure image generated by the sensor data and the result after using the 8-neighborhood correlation pixel method. (**c**) is the result of median filtering, (**d**) is the result of Gaussian filtering and (**e**) is the result of mean filtering.

For a grayscale image, the different value of each point shows a different brightness, that is, 0 means the darkest and 255 means the brightest. The larger the pressure on the sensing point, the brighter the pixels in the image. According to the grayscale image generated from the sensor data, the distribution of plantar pressure can be judged intuitively. However, it is inevitable that there will be some noise in the obtained data, which mainly includes equipment measurement noise, transmission noise, etc. In general, the noise data exist in the form of some isolated points, which can be observed intuitively after the two-dimensional matrix is imaged, as shown in Figure 4a. For the convenience of display and explanation, the pressure data of each point are flipped, that is, the darker the area in the image, the larger the pressure value it has and the higher the gray value.

#### 2.3.1. Isolated Point Removal Using an 8-Neighborhood Correlation Pixel Method

In order to get accurate results, it is necessary to remove the noise. In this work, the proposed 8-neighborhood correlation pixel method is used to remove the interference of isolated points, instead of a traditional median filter, Gaussian filter or other filters, which can obtain higher measurement accuracy. The left part of Figure 4b is the original plantar pressure image generated by the sensor data; it can be seen that there are many isolated noise points. Figure 4a shows the definition of the 8-neighborhood correlation pixel; we take the Pixels 1 to 8 around Pixel 0 as the 8-neighborhood correlation pixels of Pixel 0. The method is if one pixel’s corresponding 8-neighborhood correlation pixels gray value are all 0, then this pixel is a noise point; its value is set to zero and its 8-neighborhood correlation pixels’ gray values are left unchanged. Then, all of the 2288 pixels are processed in the same way. The resulting image from using proposed 8-neighborhood correlation pixel method is shown in the right part of Figure 4b,d and is the result of median filtering; Figure 4e,f shows the results of Gaussian filtering and mean filtering, respectively. It can be observed that the Gauss filter and mean filter both make the image blurred and make the value of the foot arch disappear, which will make the measured arch index smaller. Median filtering does not blur the image, but more effective points at the foot arch are filtered out, so the deviation of the arch index based on this will be larger. As shown in Figure 4c, the designed 8-neighborhood correlation pixel method can not only remove the isolated points but also ensure that the effective arch data points are not removed, thus ensuring the accuracy of the measurement.

#### 2.3.2. Toe Data Removal with the Row Element Association Algorithm

As mentioned above, the measurement of the arch index is based on the calculation of the area of each part of the foot sole, excluding the area of the toes, so the accurate removal of the corresponding data of the toes is the premise of the automatic and accurate measurement of the foot arch index. In this work, the row element association algorithm is proposed to remove the toes’ data. Using this algorithm, the value of the toes in the matrix can be set to 0, while the value of the foot sole is not be affected. The range of pixels processed by this method is 0 to 15 rows and 0 to 52 columns. The reason for choosing such a range is that when the toes of the subjects are flush with Row 0, according to the large number of actual measurements, the range of 15 rows covers all the pressure area of the toes but does not include the foot sole part.

The size of each sensing point of the used flexible membrane pressure sensor is 0.7 cm × 0.7 cm. When one toe acts on the sensor, the maximum number of effective sensing points in one row is no more than six consecutive points (generally, the largest thumb width is no more than 3.0 cm and less than the size of five sensing points; here, we chose six points of a size of 4.2 cm, leaving some allowance), that is, in the corresponding image—the effective toe data—at most six occupy consecutive pixel points in one row. However, the number of consecutive sensing points covered by the foot sole is generally about 10 in one row. According to this difference, using the row element association method can distinguish the toes’ data and remove them, leaving only the information of the foot sole.

The details of the methods are as follows. Evaluating each pixel value from Row 0 to Row 15, if the number of continuous effective pixel points is less than or equal to 6, then these points’ gray values are set to zero. In each row, there are six possible situations when the number of consecutive effective pixels is less than or equal to 6:

(1) For the row with 6 continuous effective pixel points, on the right side of the first effective pixel point, there are still 5 continuous effective pixel points. On the left side of the first effective pixel point, the pixel point’s gray value is 0. This can be expressed as Equation (3).

(2) For the row with 5 continuous effective pixel points, on the right side of the first effective pixel point, there are still 4 continuous effective pixel points. On the left side of the first effective pixel point, the pixel point’s gray value is 0. This can be expressed as Equation (4).

**Figure 5:**
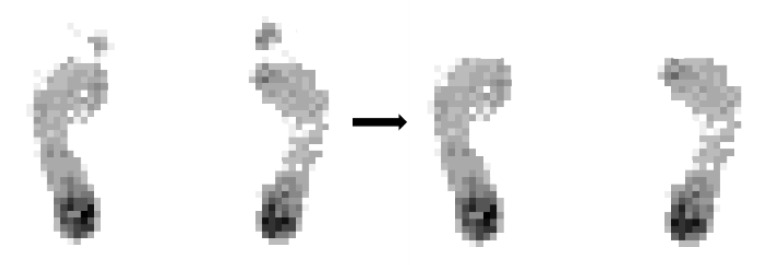
Result of removing the toe part data.

Similarly, the rest of the situations are expressed in Equations (5)–(8), respectively, where *i* is the row coordinate, *j* is the column coordinate, and *p(i,j)* is the gray value. After removing the data that satisfy the equations, the result shown in Figure 5 is produced. It can be seen that the pressure data of the toe part are effectively filtered.

(3) $$\begin{array}{l} \sum_{i=0}^{15}\sum_{j=1}^{51}(P(i,j-1)=0\;\&\;P(i,j)\geq 1\;\&\;P(i,j+1)\geq 1\;\&\;P(i,j+2)\geq 1\;\&\;P(i,j+3)\geq 1\;\&\;P(i,j+4)\geq 1\\ \&\;P(i,j+5)\geq 1), \end{array}$$

(4) $$\sum_{i=0}^{15}\sum_{j=1}^{51}(P(i,j-1)=0\;\&\;P(i,j)\geq 1\;\&\;P(i,j+1)\geq 1\;\&\;P(i,j+2)\geq 1\;\&\;P(i,j+3)\geq 1\;\&\;P(i,j+4)\geq 1),$$

(5) $$\sum_{i=0}^{15}\sum_{j=1}^{51}(P(i,j-1)=0\;\&\;P(i,j)\geq 1\;\&\;P(i,j+1)\geq 1\;\&\;P(i,j+2)\geq 1\;\&\;P(i,j+3)\geq 1),$$

(6) $$\sum_{i=0}^{15}\sum_{j=1}^{51}(P(i,j-1)=0\;\&\;P(i,j)\geq 1\;\&\;P(i,j+1)\geq 1\;\&\;P(i,j+2)\geq 1),$$

(7) $$\sum_{i=0}^{15}\sum_{j=1}^{51}(P(i,j-1)=0\;\&\;P(i,j)\geq 1\;\&\;P(i,j+1)\geq 1),$$

(8) $$\sum_{i=0}^{15}\sum_{j=1}^{51}(P(i,j-1)=0\;\&\;P(i,j)\geq 1).$$

#### 2.3.3. Binarization of the Effective Data of the Foot Sole

**Figure 6:**
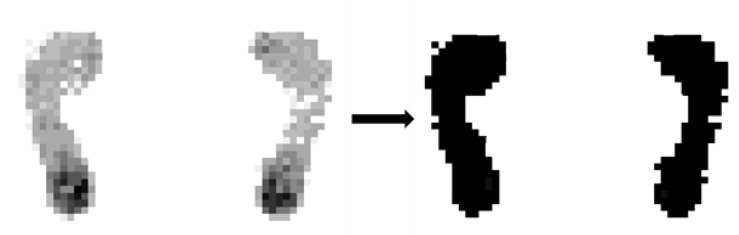
Binarization of the result of the effective data of the foot sole.

The arch index in this work is calculated based on the ratio of the arch pressure area, so it is not necessary to know the real pressure value of each point. Therefore, in order to facilitate the subsequent calculation, we binarize the effective pressure value of the foot sole to obtain a binary image, as shown in Figure 6 [29]. The gray value of the corresponding effective pixel is set to 255 as expressed in Equations (9) and (10).

(9) $$\sum_{i=0}^{43}\sum_{j=0}^{51}P(i,j)=255,\mathrm{if}(P(i,j)>0)$$

(10) $$\sum_{i=0}^{43}\sum_{j=0}^{51}P(i,j)=0,\mathrm{if}(P(i,j)=0)$$

#### 2.3.4. Automatic Recognition and Location of Foot Endpoints

**Figure 7:**
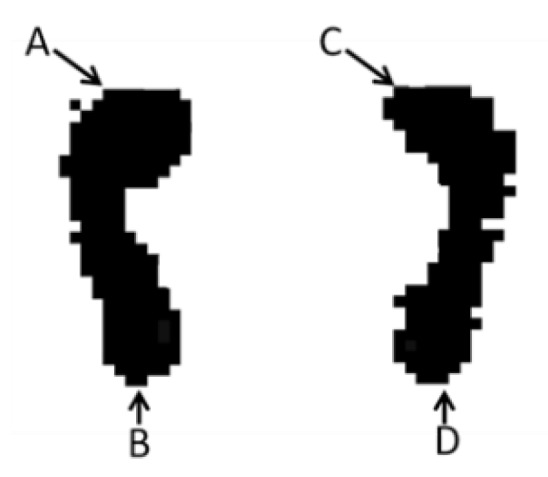
Endpoints of the left and right soles.

In order to realize the automatic measurement of the arch index, an important premise is to know the foot length, to extract the key endpoints of the left and right soles. Thus, for the left sole, from left to right, from top to bottom, all pixel points are traversed in turn and the location of the first non-zero pixel point is recorded as *A (i, j)*, and the coordinates of the last non-zero point are recorded as *B (i, j)*, as expressed as Equations (11) and (12). In the same way, the right sole’s endpoints *C (i, j)* and *D (i, j)* can be also obtained, as expressed as Equations (13) and (14), respectively, where *i* and *j* are the endpoints’ row coordinate and column coordinate, as shown in Figure 7.

(11) $$A(i_{A},j_{A})=\mathrm{first}(\sum_{i=0}^{43}\sum_{j=0}^{25}P(i,j)=255)$$

(12) $$B(i_{B},j_{B})=\mathrm{last}(\sum_{i=0}^{43}\sum_{j=0}^{25}P(i,j)=255)$$

(13) $$C(i_{C},j_{C})=\mathrm{first}(\sum_{i=0}^{43}\sum_{j=25}^{51}P(i,j)=255)$$

(14) $$D(i_{D},j_{D})=\mathrm{last}(\sum_{i=0}^{43}\sum_{j=25}^{51}P(i,j)=255)$$

### 2.4. Calculation of Foot Arch Index

After the endpoint coordinates are obtained, the length of the foot can be calculated as shown in Equations (15) and (16). $\alpha_{1},\alpha_{2}$ are the lengths of the left and right feet, respectively.

(15) $$\alpha_{1}=i_{B}-i_{A}$$

(16) $$\alpha_{2}=i_{D}-i_{C}$$

The corresponding effective pressure area can be calculated as follows:

(17) $$S_{1}=(\sum_{i=i_{A}}^{i_{A}+\alpha_{1}/3}\sum_{j=0}^{25}P(i,j)=255)$$

(18) $$S_{2}=(\sum_{i=i_{A}+\alpha_{1}/3}^{i_{A}+2*\alpha_{1}/3}\sum_{j=0}^{25}P(i,j)=255)$$

(19) $$S_{3}=(\sum_{i=i_{A}+2*\alpha_{1}/3}^{i_{A}+\alpha_{1}}\sum_{j=0}^{25}P(i,j)=255)$$

(20) $$S_{4}=(\sum_{i=i_{B}}^{i_{B}+\alpha_{2}/3}\sum_{j=25}^{51}P(i,j)=255)$$

(21) $$S_{5}=(\sum_{i=i_{B}+\alpha_{2}/3}^{i_{B}+2*\alpha_{2}/3}\sum_{j=25}^{51}P(i,j)=255)$$

(22) $$S_{6}=(\sum_{i=i_{B}+2*\alpha_{2}/3}^{i_{B}+\alpha_{2}}\sum_{j=25}^{51}P(i,j)=255)$$

Finally, according to Equation (1), the foot arch index can be expressed as

left foot arch index, $\beta_{\mathrm{left}}$:

(23) $$\beta_{\mathrm{left}}=s_{2}/(s_{1}+s_{2}+s_{3})$$

Right foot arch index, $\beta_{\mathrm{right}}$:

(24) $$\beta_{\mathrm{right}}=s_{5}/(s_{4}+s_{5}+s_{6})$$

## 3. Prototype Design and Experiment

In this part, we build the hardware and software environment of the prototype, and a series of contrast experiments are carried out to verify the feasibility and validity of the proposed method.

### 3.1. Construction of Software and Hardware Environment

**Figure 8:**
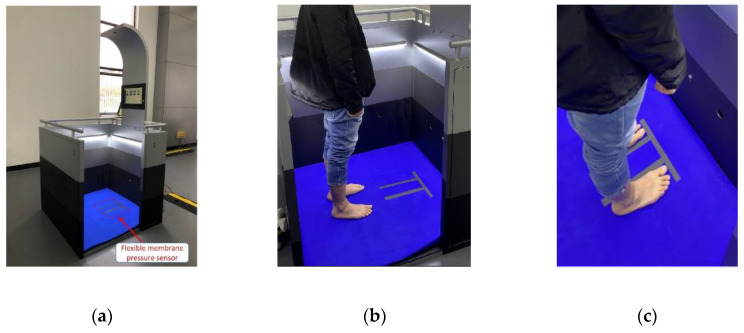
Prototype of foot arch index measurement. (**a**) is the designed prototype, and (**b**) and (**c**) show the correct test position.

The software environment is based on Windows 7 64-bit operating system, the software integration environment is VS-2017, and the image processing software is OpenCV-3.0. The processor is AMD FX-8350, and the memory size is 16 G with a corresponding frequency 1600 MHZ, and the R9-390x-8G is used as the graphics card. The flexible membrane pressure sensor is placed under the carpet, as shown in Figure 8a. The collected pressure data are transmitted to the computer through USB; the data refresh frequency can be adjusted flexibly according to the specific requirements. Figure 8a shows the designed prototype. The tester has to stand in the specified test area, as shown in Figure 8b,c.

### 3.2. Contrast Experiments

To evaluate the accuracy and repeatability of the proposed method, the contrast experiments are carried out, including the manual foot arch index measurement and the proposed automatic measurement. The result of the manual measurement is regarded as the reference value, while the automatic measurement result is the value to be verified.

In the experiment, three subjects were selected with a high arch foot (male, 26 years old), normal foot (male, 34 years old) and flat foot (male, 28 years old), respectively. For each subject, 10 manual measurements and 10 automatic measurements were carried out at different times, respectively. In total, 60 (3 × 2 × 10) groups of data were collected. The following is the detailed measurement process.

#### 3.2.1. Manual Measurement

**Figure 9:**
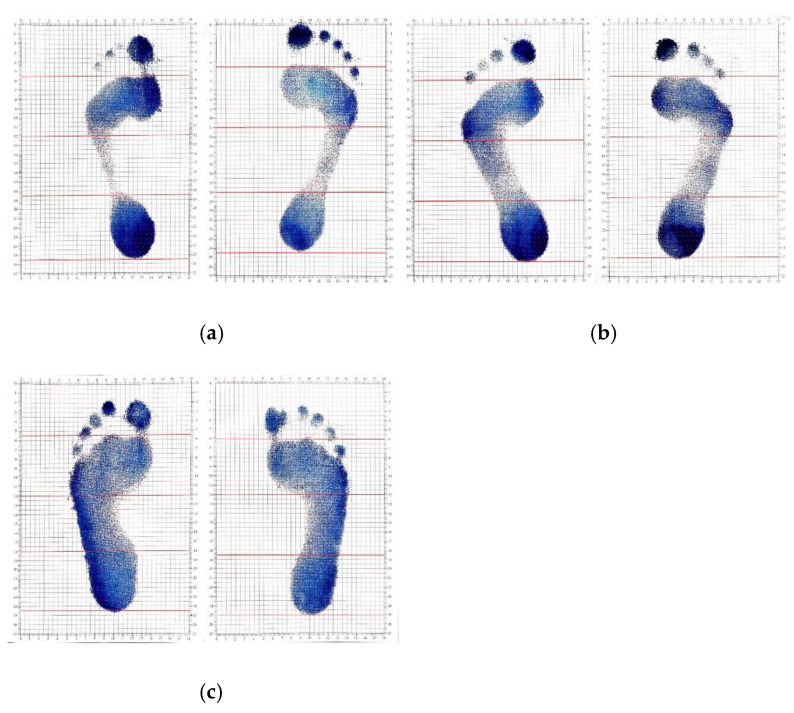
Footprints obtained by manual measurement. (**a**) High arch foot. (**b**) Normal foot. (**c**) Flat foot.

The manual measurement is to smear some blue ink on the feet of the subjects, and then the subjects stand naturally on the graph paper to make the footprints, which are shown in Figure 9. Then, the number of effective squares occupied by the footprints is manually counted. When the blue area is less than half of one square, it is omitted, and when the blue area is more than half of the square, is considered as effective. Thus, according to Equations (23) and (24), the corresponding arch index can be obtained.

When the measured value is less than or equal to 0.2, it means a high arch foot. When the measured value is between 0.2 and 0.26, it indicates that the subject has normal feet. If the measured value is greater than or equal to 0.26, it indicates that the subject is a flat foot patient [11]. The measurement results are shown in Table 1, Table 2 and Table 3.

#### 3.2.2. Automatic Measurement with Proposed Method

**Figure 10:**
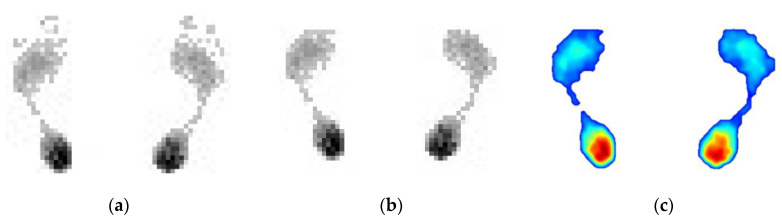
The results of automatic processing based on the proposed method for the high arch feet. (**a**) is the original plantar pressure image of the high arch feet, (**b**) is result after using the proposed method and (**c**) is the interpolation color image of (**b**).

**Figure 11:**
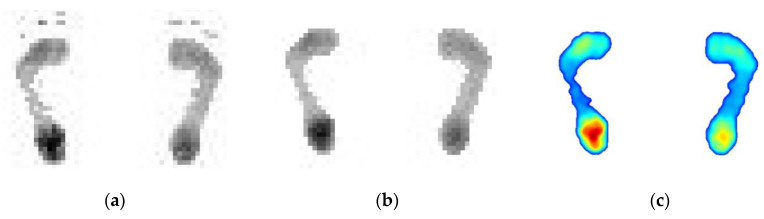
The results of automatic processing based on the proposed method for the normal feet. (**a**) is the original plantar pressure image of the normal feet, (**b**) is result after using the proposed method and (**c**) is the interpolation color image of (**b**).

**Figure 12:**
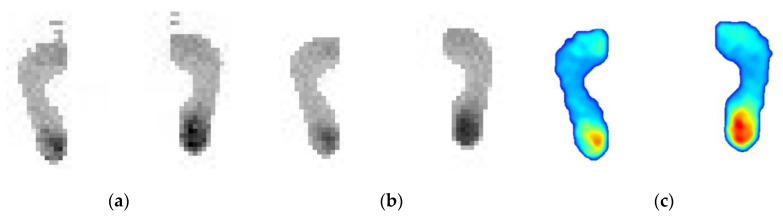
The results of automatic processing based on the proposed method for the flat feet. (**a**) is the original plantar pressure image of the flat feet, (**b**) is result after using the proposed method and (**c**) is the interpolation color image of (**b**).

Using the designed prototype shown in Figure 8, the results of the automatic processing based on the proposed method are shown in Figure 10. Figure 10a is the original plantar pressure image of the high arch feet. Figure 10b is the result after using the proposed 8-neighborhood correlation pixel filter and row element association algorithm, and the interpolation color image is shown in Figure 10c. Similarly, the test images of the normal foot and flat foot are shown in Figure 11 and Figure 12, respectively. As with the manual measurement, each subject was tested 10 times. The calculated arch index results are also shown in Table 1, Table 2 and Table 3.

## 4. Discussion

The contrast experiment results are shown in the following tables. Within the error range allowed, both the results of the manual measurement and proposed automatic measurement methods have some fluctuations. In the manual measurement, the mean values for the high arch foot, normal foot and flat foot are 0.15, 0.18; 0.25, 0.25 and 0.28, 0.30, respectively. The mean values of the proposed automatic measurement results are 0.15, 0.17; 0.25, 0.24 and 0.28, 0.29, respectively.

**Figure 13:**
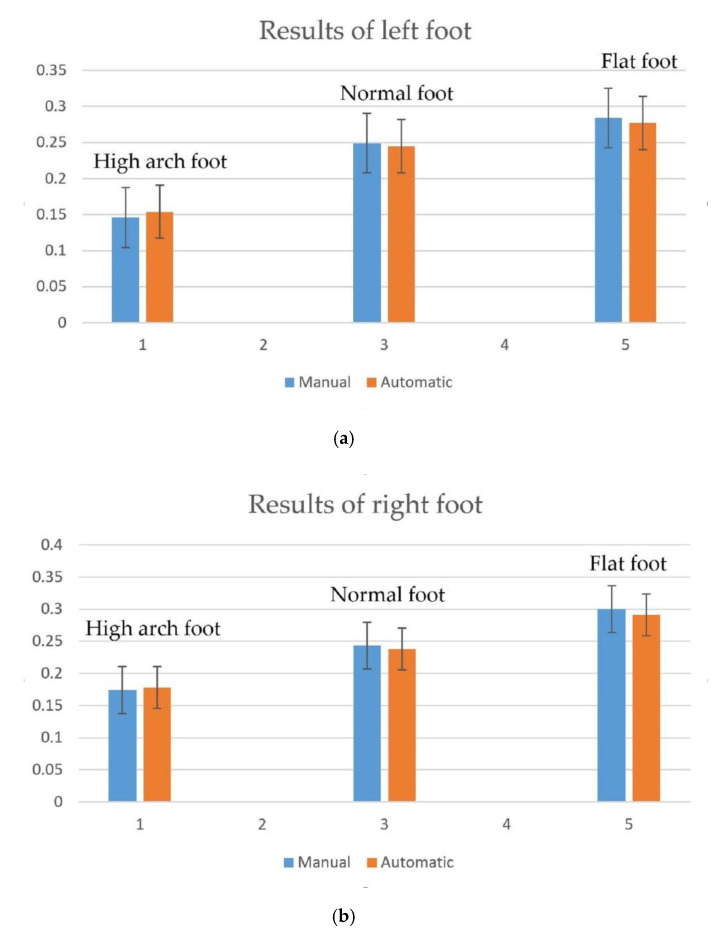
Comparison curve for the two measurement methods. (**a**) Comparison of left foot results. (**b**) Comparison of right foot results.

We calculated the average absolute deviation (AVEDEV) and standard deviation (STDEV) of each subject’s left foot and right foot to evaluate the accuracy and repeatability. As shown in Table 4, the average AVEDEV of the manual and automatic measurements are 0.0071 and 0.0069, respectively. The average STDEV are 0.0093 and 0.0090, respectively. This means that the dispersion of data is low, and to some extent, the proposed automatic measurement method has a higher repeatability. In addition, the coefficient of variation (CV) is now widely used to express precision and repeatability; the calculated average CVs are 0.0440 and 0.0408, which also verifies the correctness and reliability of the method. From Figure 13, the test results of the two methods are observed to be almost the same. They can all distinguish different foot types clearly. Thus, we can consider that the proposed automatic foot arch measurement method could replace manual measurement and meet the accuracy requirements of medical foot arch index measurement. Of course, it should be noted that during the measurement, the subjects cannot tilt left or right at will, and they need to stand naturally and keep still, so that the data obtained will be accurate and have the reference value of actual medical diagnosis; otherwise, there will be a large deviation.

## 5. Conclusions

In this work, a fast and accurate automatic foot arch index measurement method based on a flexible membrane pressure sensor is proposed. The 8-neighborhood correlation pixel method is used to remove the isolated noise points, which ensures that the effective arch data points are not removed. The row element association algorithm is proposed to remove the toes’ corresponding data without affecting the data of the foot sole, which provides a guarantee for the subsequent accurate calculation of the area. Then, by traversing the row elements, the endpoints of the foot to calculate the area of each part of the foot are found. The prototype is developed to validate the proposed method. Comparing with the manual measurement results, the average absolute deviation, standard deviation and coefficients of variation of the automatic measurement are 0.0069, 0.0090 and 0.0408, respectively, while those for the manual measurement are 0.0071, 0.0093 and 0.0440, respectively. It means that the dispersion of the automatic measurement results is lower and that the proposed automatic measurement method has a higher repeatability. The experimental results validate the validity and reliability of the proposed method. It is also worthwhile to point out that the designed prototype is worth being extended to actual foot arch index medical diagnosis.

## Figures and Tables

**Table 1:** Results of the two measurement methods for the high arch feet.

Foot Arch Index	Manual Measurement Results		Automatic Measurement Results	
	Left Foot	Right Foot	Left Foot	Right Foot
**Group 1**	0.14	0.17	0.15	0.18
**Group 2**	0.13	0.19	0.15	0.18
**Group 3**	0.15	0.18	0.14	0.19
**Group 4**	0.14	0.16	0.16	0.17
**Group 5**	0.14	0.15	0.15	0.18
**Group 6**	0.16	0.17	0.16	0.16
**Group 7**	0.15	0.17	0.17	0.18
**Group 8**	0.14	0.18	0.15	0.19
**Group 9**	0.15	0.18	0.16	0.17
**Group 10**	0.16	0.19	0.15	0.18
**Average**	0.146	0.174	0.154	0.178
**AVDEV**	0.0080	0.0100	0.0068	0.0068
**STDEV**	0.0097	0.0126	0.0084	0.0092
**CV**	0.0662	0.0727	0.0548	0.0516

**Table 2:** Results of the two measurement methods for the normal feet.

Foot Arch Index	Manual Measurement Results		Automatic Measurement Results	
	Left Foot	Right Foot	Left Foot	Right Foot
**Group 1**	0.23	0.25	0.23	0.24
**Group 2**	0.26	0.25	0.25	0.24
**Group 3**	0.25	0.23	0.24	0.24
**Group 4**	0.25	0.25	0.25	0.24
**Group 5**	0.26	0.24	0.23	0.23
**Group 6**	0.25	0.25	0.25	0.24
**Group 7**	0.24	0.24	0.24	0.23
**Group 8**	0.25	0.23	0.25	0.23
**Group 9**	0.25	0.25	0.26	0.25
**Group 10**	0.25	0.24	0.25	0.24
**Average**	0.249	0.243	0.245	0.238
**AVDEV**	0.0056	0.0070	0.0080	0.0048
**STDEV**	0.0088	0.0082	0.0097	0.0063
**CV**	0.0352	0.0339	0.0397	0.0266

**Table 3:** Results of the two measurement methods for the flat feet.

Foot Arch Index	Manual Measurement Results		Automatic Measurement Results	
	Left Foot	Right Foot	Left Foot	Right Foot
**Group 1**	0.28	0.3	0.27	0.29
**Group 2**	0.27	0.29	0.28	0.29
**Group 3**	0.28	0.3	0.26	0.3
**Group 4**	0.29	0.3	0.28	0.31
**Group 5**	0.29	0.31	0.27	0.29
**Group 6**	0.29	0.3	0.29	0.27
**Group 7**	0.28	0.31	0.29	0.28
**Group 8**	0.28	0.31	0.28	0.29
**Group 9**	0.29	0.3	0.27	0.3
**Group 10**	0.29	0.28	0.28	0.29
**Average**	0.284	0.3	0.277	0.291
**AVDEV**	0.0060	0.0060	0.0076	0.0074
**STDEV**	0.0070	0.0094	0.0095	0.0110
**CV**	0.0246	0.0314	0.0342	0.0378

**Table 4:** Deviation assessment.

	Manual Measurement Results			Automatic Measurement Results		
	AVEDEV	STDEV	CV	AVEDEV	STDEV	CV
**1**	0.0080	0.0097	0.0662	0.0068	0.0084	0.0548
**2**	0.0100	0.0126	0.0727	0.0068	0.0092	0.0516
**3**	0.0056	0.0088	0.0352	0.0080	0.0097	0.0397
**4**	0.0070	0.0082	0.0339	0.0048	0.0063	0.0266
**5**	0.0060	0.0070	0.0246	0.0076	0.0095	0.0342
**6**	0.0060	0.0094	0.0314	0.0074	0.0110	0.0378
**Average**	0.0071	0.0093	0.0440	0.0069	0.0090	0.0408
